# Analysis of Dark Current in BRITE Nanostellite CCD Sensors †

**DOI:** 10.3390/s18020479

**Published:** 2018-02-06

**Authors:** Adam Popowicz

**Affiliations:** Institute of Automatic Control, Silesian University of Technology, Akademicka 16, 44-100 Gliwice, Poland; apopowicz@polsl.pl

**Keywords:** CCD defects, space telescopes, nano-satellites

## Abstract

The BRightest Target Explorer (BRITE) is the pioneering nanosatellite mission dedicated for photometric observations of the brightest stars in the sky. The BRITE charge coupled device (CCD) sensors are poorly shielded against extensive flux of energetic particles which constantly induce defects in the silicon lattice. In this paper we investigate the temporal evolution of the generation of the dark current in the BRITE CCDs over almost four years after launch. Utilizing several steps of image processing and employing normalization of the results, it was possible to obtain useful information about the progress of thermal activity in the sensors. The outcomes show a clear and consistent linear increase of induced damage despite the fact that only about 0.14% of CCD pixels were probed. By performing the analysis of temperature dependencies of the dark current, we identified the observed defects as phosphorus-vacancy (PV) pairs, which are common in proton irradiated CCD matrices. Moreover, the Meyer-Neldel empirical rule was confirmed in our dark current data, yielding $E_{MN}=24.8$ meV for proton-induced PV defects.

## 1. BRITE-Constellation

The BRight Target Explorer (BRITE) is a constellation of six nanosatelites launched in 2013–2014, which are dedicated to high-precise photometry of the brightest stars in blue (b) and red (r) filters. The mission is a collaboration between Canada, Austria and Poland. Each of these countries has two satellites: UniBRITE (UBr) and BRITE Austria (BAb) are Austrian satellites, BRITE Toronto (BTr) and BRITE Montreal (failed to communicate after launch) are Canadian satellties, BRITE Lem (BLb) and BRITE Heweliusz (BHr) are Polish. The satellites are only 20×20×20 cm in size and 7 kg in weight, which makes them the smallest satellites performing accurate photometry from space. The spacecraft were designed using the Generic Nanosatellite Bus by the University of Toronto and Space Flight Laboratory. They include 3-axis, reaction wheel stabilization which is performed by the Attitude Determination and Control System (ADCS) utilizing sun sensors, magnetometers and a star-tracker. More detailed descriptions of the satellite construction, testing, commissioning and operations can be found in our papers [1,2].

The heart of the satellites is the monochromatic, interline CCD sensor, Kodak KAI-11002M, which is attached to a custom 5-element (4-element in BHr) lens system, which allows observation of a wide, 24${}^{\circ }$ in diameter, field of view. Basic characteristics of employed full-frame image sensors are listed in Table 1. The combination of large-area image sensor and a dedicated lens system allowed for maximizing the number of stars measured simultaneously in a single field. This was also a compromise between the satellite size constraints, the required photometric precision, the pixel and chip size, and the number of targets monitored during the mission’s expected lifetime.

In this paper we assess the progress of degradation of CCD matrices installed on-board BRITE satellites during almost four years of their in-orbit work. Due to the lack of thermal stabilization and varying exposure times (1–5 s, depending on the intensities of stars included in the field), such analysis is not a trivial task and it requires applying several steps of image processing and proper normalization of the data. Moreover, the analysis can utilize information only from a very small portion of the CCD arrays (i.e., only several tiny subrasters per field) and at unstabilized temperatures, which are dependent on the observed field.

## 2. Imaging with BRITE Satellites

**Figure 1:**
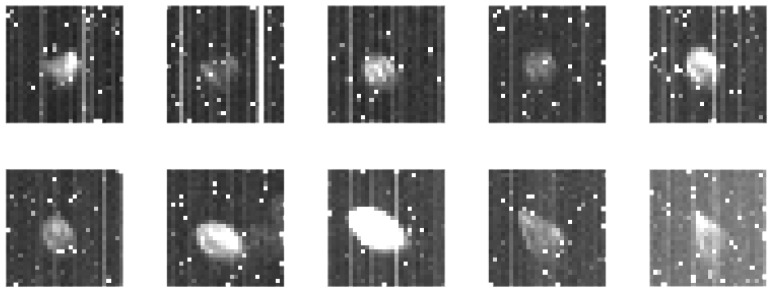
Examples of subrasters from an exposure taken by the BAb satellite during observations of a field in Vela-Puppis constellations. Different shapes of profiles are due to the combination of defocusing and abberations in the optical path.

To make photometric measurements possible, the optical system of the BRITE satellites is intentionally defocussed, so that the stellar profiles are spread over several tens of pixels. During normal operations, only small parts of the image (so-called subrasters), around selected targets, are transferred to ground stations, where the photometric measurements are performed by special processing procedures [3,4]. Several examples of 28 × 28-pixel subrasters with stellar profiles from various parts of the image sensor are presented in Figure 1.

As one can see in Figure 1, the stellar profiles are accompanied by significant noise in the form of bright pixels and columns. While the former is the result of dark current generation in silicon defects inflicted by energetic particles (protons) impacting the CCD sensor, the latter appears due to the dark current generated during the matrix readout in the vertical transferring register (VCCD –> see more information about the sensor in the datasheet (http://www.onsemi.com/pub/Collateral/KAI-11002-D.PDF)). Since both types of noise increase with temperature and the installed sensors are not thermally stabilized, special techniques of satellite orientation are undertaken to reduce unwanted heat. Nevertheless, the temperature of the sensors is usually within 10–40 ${}^{\circ }$C, depending on which field is observed. Three of BRITEs (UBr, BAb and BLb) do not house any shielding against radiation, while the remaining two are equipped with Borotrone (BHr) and tungsten (BTr) shielding, respectively. Importantly, in BTr, due to the lack of space and no chance for re-design, only the aluminum CCD header tray was exchanged with one made of tungsten (2 mm thick). In BHr, which has a different optical design, it was possible to install light-weight hydrogen-rich polyethylene (Borotron) shielding (10 mm thick).

**Figure 2:**
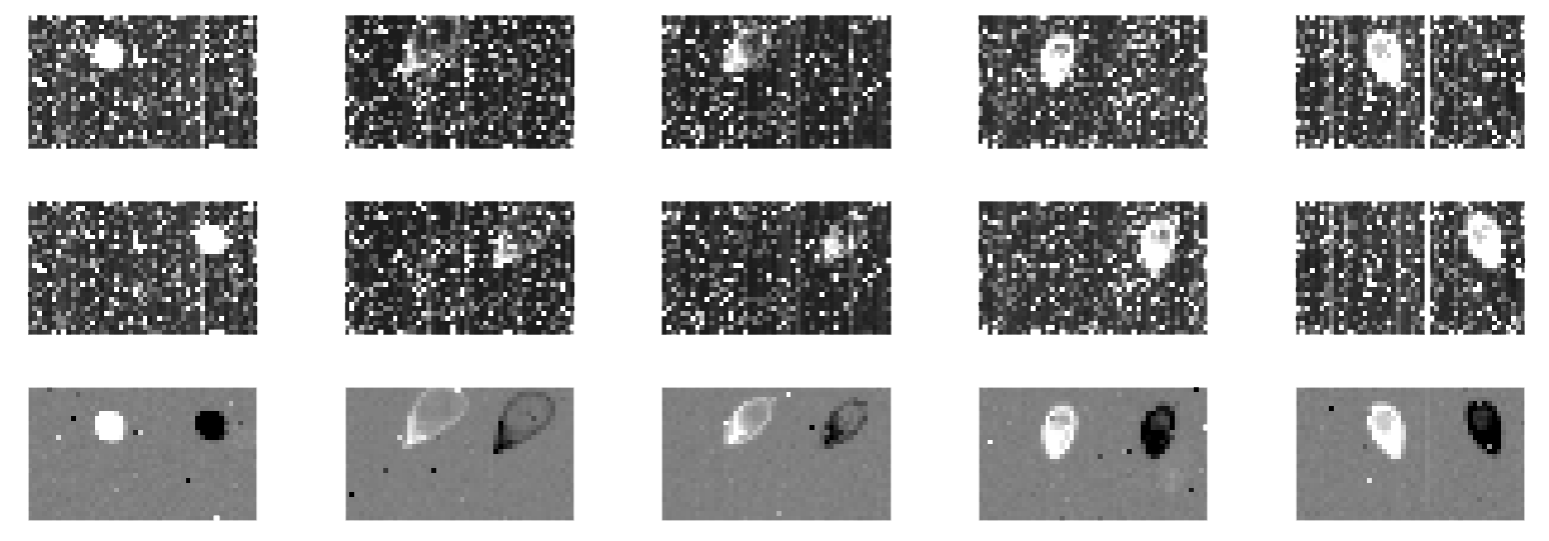
Examples of the efficient reduction of impulsive noise thanks to the chopping mode. The two upper rows show five subrasters from two consecutive measurements. The bottom row shows fully reduced, nearly noiseless differences between the respective upper two images. Data collected by BLb in Cygnus field at 39 ${}^{\circ }$C.

The BRITE constellation was initially operated in so-called stare mode, in which a satellite orientation was stabilized so that stellar profiles experienced only slight (usually sub-pixel) shifts. Unfortunately, due to the increase in observed noise and the accompanying decrease of photometric quality, a so-called chopping mode was introduced and is currently employed by all satellites. In this mode, a satellite is moved alternately by several arc minutes (from frame to frame) so that the stellar profile is swung between two positions. This allows for operating with difference images (i.e., subtraction of two consecutive images), in which the offsets related to the dark current are virtually eliminated. Additionally, in the chopping mode, the subrasters have to be elongated horizontally (24 × 48 pixels) to allow for safe placement of the stellar profile well within the subraster. This is required due to some imperfections of satellite stabilization in both chopping positions. An example of the utilization of the chopping mode is presented in Figure 2. A few black or white spots visible in difference images (bottom row) are the result of occasional sudden change of dark current generation, a phenomenon which is called random telegraph signals (RTS) and was already reported in proton-irradiated sensors [5].

## 3. Data Analysis

### 3.1. CCD Image Processing

As an objective measure of the amount of a sensor’s degradation, we selected the mean dark charge ($I_{d}$) generated in the photosensitive part of a pixel. However, since the dark charge is collected both during the exposure and in the readout phase, the former depends on the exposure time while the latter does not. Therefore, the dark current generated during the readout, visible as offsets in columns, has to be compensated to reveal only the thermal generation in photosensitive sites. This type of dark current appears due to the thermal generation of electrons within the defects induced in the transferring part of a pixel (i.e., in VCCD register). As a result, a slight intensity offset is added to all charge pockets transferred through a defective cell during the readout.

**Figure 3:**
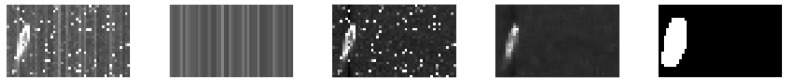
An example of the result of the compensation of readout dark current and detection of a stellar profile. From the left: original image, column medians, image after column compensation, outcome of median filtering, detected stellar profile.

To this end, initially the median intensity is calculated in each column of a subraster. Then, the medians are subtracted from the pixels of each column, so that the column offsets are compensated. This way not only is the readout dark current calibrated, but also the charge bias appearing due to the scattered light from Moon or Earth shine is effectively removed. After such column compensation, the subraster is filtered with a 3 × 3 median filter, so that hot pixels are removed, revealing only the stellar profile. Such a median-filtered image is thresholded with 50 ADU and dilated by 2 pixels to find all the pixels covered by a stellar profile. Dilation is necessary to include some wings that are dimmer than 50 ADU but that still belong to the stellar profile. Such a value was chosen to warrant robustness against readout noise while keeping the high detection efficiency or the faintest parts of a profile. The process of column compensation of raw images is then repeated excluding the stellar profile from the calculation of column medians. The procedure is iteratively repeated until the detected profile stabilizes. This allows for unbiased estimation and reduction of column offsets. An example of the result from the steps of the routine mentioned above is shown in Figure 3.

Now we can calculate the average intensity of a pixel in the compensated image excluding pixels detected as a stellar profile. Since the offsets in columns are subtracted, the remaining positive charge is related only to the thermal generation of charge (readout noise has zero mean value thus it does not disturb the calculations). By dividing the whole collected charge by the number of investigated pixels and by the exposure time, we eventually obtain the mean dark current $I_{d}$ expressed in electrons per second per pixel.

**Figure 4:**
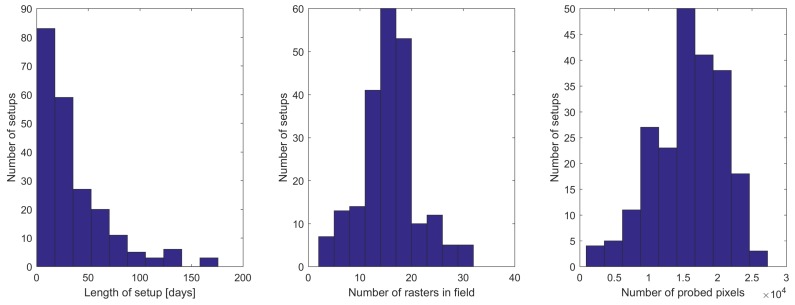
Characteristics of data utilized in the dark current analysis. From the left: length of setups in days, number of subrasters per field and number of probed pixels.

In the calculations we use all the subrasters downloaded from the satellite in a given exposure. Depending on the observed field and on the satellite, there are 3 to 32 subrasters available. On average a total number of 15,800 pixels was probed per exposure, which is only 0.14% of the total number of CCD pixels (11 million). Histograms of the number of subrasters and pixels included in the analysis are illustrated in the middle and right panel of Figure 4.

Importantly, the data collected by the satellites are divided into so-called setups, which are the data sets having the same positions of stars and sizes of subrasters. The setups were introduced to maintain consistency in BRITE data. They are changed only in case of problems with satellite stability (e.g. lack of proper guide-stars or increase of the scattered light from the Moon or Earth) and when switching between observed fields. The spread of time span covered by the setups is presented in Figure 4, on the left. While here we present only the histograms for all satellites together, the detailed distribution of the observations from individual BRITE satellites is presented in Appendix A.1 in [3].

### 3.2. Dark Current Analysis

**Figure 5:**
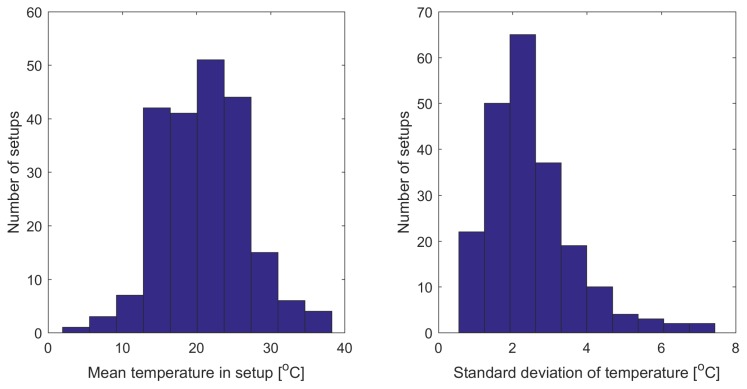
Mean CCD temperatures (**left**) and their standard daviations (**right**) for all five nanosatellites, across all setups.

**Figure 6:**
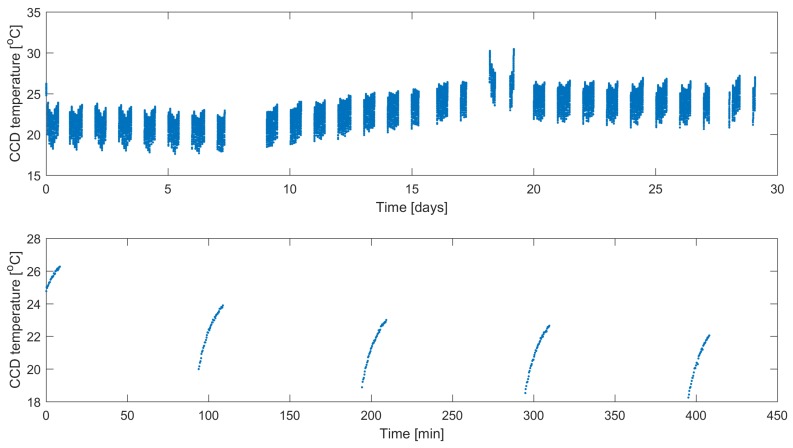
Time dependencies of CCD temperature for the BAb satellite, Orion 2016 field. Upper plot—data from whole setup; lower plot—initial five orbits.

The temperature of the BRITE CCD sensors is dependent on the orientation of the satellite relatively to the Sun; therefore, it may vary significantly between the setups. In the histograms in Figure 5, the statistics (mean and standard deviation) of temperature across all setups is presented. Moreover, the temperature usually increases within a single orbital period (≈100 min) since a satellite gradually comes out of the Earth’s shadow and the CCD starts exposures. Exemplary time dependencies of CCD temperature of the BAb satellite in the Orion2016 field are presented in Figure 6. In this case, the temperature drift within a single orbit equals approximately 4 ${}^{\circ }$C and a slight long-term trend of 5 ${}^{\circ }$C amplitude is also visible. Some transient events appear in many of the setups due to the preceding technical breaks and/or temporal maneuvers which, in the case depicted in Figure 6, resulted in the increase of heat.

The temperature variations of the BRITE CCDs are in fact very useful for the dark current analysis, since the temperature dependencies can be approximated and thus the dark current can be scaled to an arbitrary temperature. According to [6,7], the dark current should follow the Arrhenius law:

(1) $$I_{d}=G\;\exp\left(-\Delta E/kT\right),$$

where $I_{d}$ is the amount of dark charge per pixel per time interval, *G* is a constant, ΔE is the activation energy of thermal electron generation, *k* is the Boltzmann constant and *T* is the temperature in Kelvins. This relation is usually visualized by plotting the logarithm of dark charge against 1/kT (the so-called Arrhenius plot), which corresponds to the following transformation of (1):

(2) $$\log\left(I_{d}\right)=-\Delta E\frac{1}{kT}+\log\left(G\right).$$

Fitting a simple linear regression to such a dependency allows one to obtain an estimation of the activation energy ΔE of the thermal process which can lead to the identification of the type of defects. Moreover, it becomes possible to estimate the amount of dark charge at the temperature which is not available in a given setup. This enables normalization of the results to the mean dark current rate at an arbitrary temperature.

**Figure 7:**
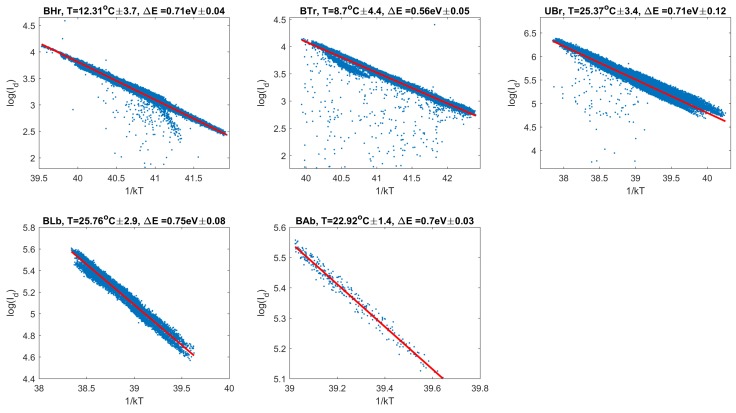
Arrhenius plots of the dark current for some setup examples. The following setups (satellites) are: Carina 2017 setup 8 (BHr), Vela Pictor 2016 setup 4 (BTr), Cassiopeia 2016 setup 2 (UBr), Cygnus Lyra 2016 setup 3 (BLb) and Orion 2016 setup 1 (BAb). Red lines indicate a robust linear fit to data points.

The correctness of Equation (1) was confirmed in our data. Clearly linear dependencies appeared when the logarithm of dark charge was plotted against 1/kT. In Figure 7, we present the results from setup examples collected by BRITE satellites in various temperature conditions and with different numbers of measurements. With these data, robust linear fitting (iteratively reweighted least squares with a bisquare weighting function, [8]) was performed to estimate the activation energy ΔE and the factor *G*.

## 4. Results and Discussion

**Figure 8:**
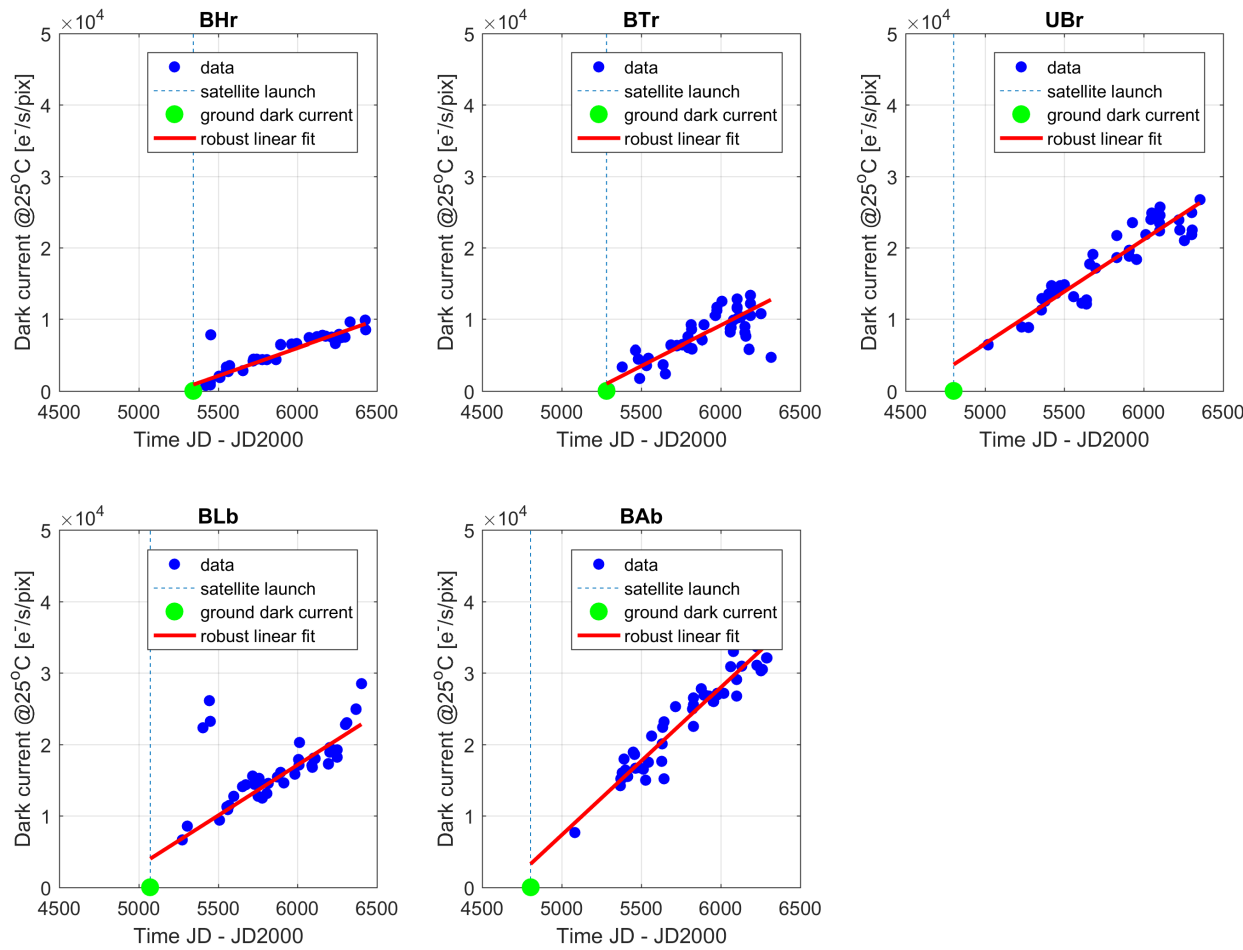
Time dependencies of dark current at 25 ${}^{\circ }$C in BRITE CCDs for all five nanosatellites and almost four years of being in orbit. Dashed vertical line indicates the launch date and the green point shows the ground-based dark current. Time is expressed in days elapsed since the epoch 2000.0. Red line is the robust linear fit to the data points (excluding the green point).

Using the liner relation between temperature and dark current as presented on the Arrhenius plots, it is possible to calculate the amount of dark current at 25 ${}^{\circ }$C across the mission lifetime and for all five CCDs. For this particular temperature, the ground based tests indicated that the dark current generation rate equals 21 [$e^{-}s^{-1}$pixel${}^{-1}$] (see Table 4 in [1]). The gradual increase of the dark current in each of the five nanosatellites can be observed in Figure 8. The red line shows a robust linear fit to the data, while the blue dashed vertical line indicates the launch date. The derived increase of the dark current expressed as a growth of the number of electrons per second per pixel at three temperatures (15 ${}^{\circ }$C, 25 ${}^{\circ }$C and 35 ${}^{\circ }$C), covering typical thermal conditions of the BRITE CCDs, is listed in Table 2.

It is apparent that additional shielding installed in the BHr satellite resulted in significant limitation of the rate of growth of the defects induced in the CCD. The growth of the dark current is nearly twice as small when compared with UBr and BLb and almost three times as small when compared with BAb. The tungsten CCD tray installed in BTr was not so successful; however, it still allowed for absorbing nearly 25% of the damage. It is not clear why the BAb satellite shows 50% larger dark current growth rate when compared to UBr and BLb. All unshielded satellites are operated the same way, stay in orbits of similar height, and share the same optical and mechanical design.

An interesting observation can be made when comparing the starting point of the dark current with that expected from the linear fit. Clearly, unshielded satellites were damaged during the launch, so that the dark current generation increased immediately from 21 to approximately 4000 $e^{-}s^{-1}{\mathrm{pix}}^{-1}$ at 25 ${}^{\circ }$C. Such a growth is not present in the shielded satellites, which implies that both shielding solutions successfully protected CCDs from energetic particles created while launching a satellite into orbit.

**Figure 9:**
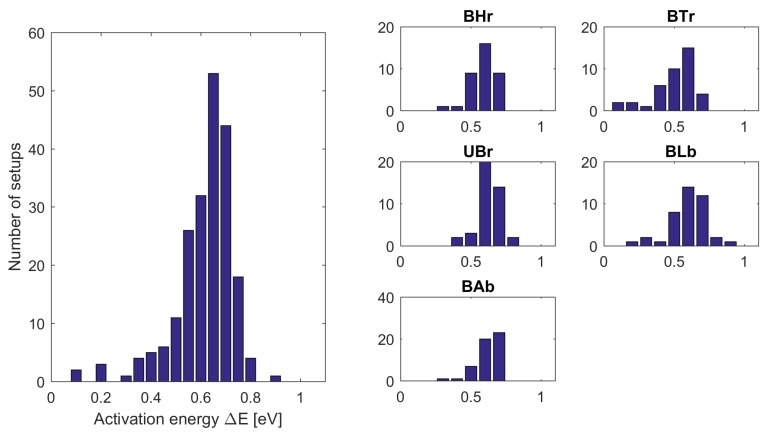
Histograms of the activation energy obtained from the dark current analysis. On the left side: collective histogram for all satellites; on the right side: individual histograms for each satellite.

The temperature dependencies of the dark current permitted the estimation of the activation energy of the thermal process in the CCD pixels. The histograms of ΔE for all setups collectively and for each satellite separately are exposed in Figure 9 on the left and right side, respectively. The median ΔE across all CCDs equals 0.68 eV and does not differ between sensors. This is very close to the activation energy of a phosphorus-vacancy (PV) dipole, which is 0.70 eV (i.e., 0.44 eV below the silicon conduction band—see chapter 8 in [9] or [10]). The induction of PV defects was also reported by many authors investigating proton irradiated CCD sensors [11,12,13].

According to the observations made in [7], the factor *G* obeys the Meyer-Neldel rule (MNR) [14], which is an experimental rule still not fully understood. This means that *G* can be expressed as follows:

(3) $$G=\bar{G}\exp\left(\Delta E/E_{MN}\right),$$

where $\bar{G}$ and $E_{MN}$ are positive MNR constants. While the former can vary between pixels and in time, since it is related to the number of defects induced in a pixel, the latter should be constant for a given type of defect.

**Figure 10:**
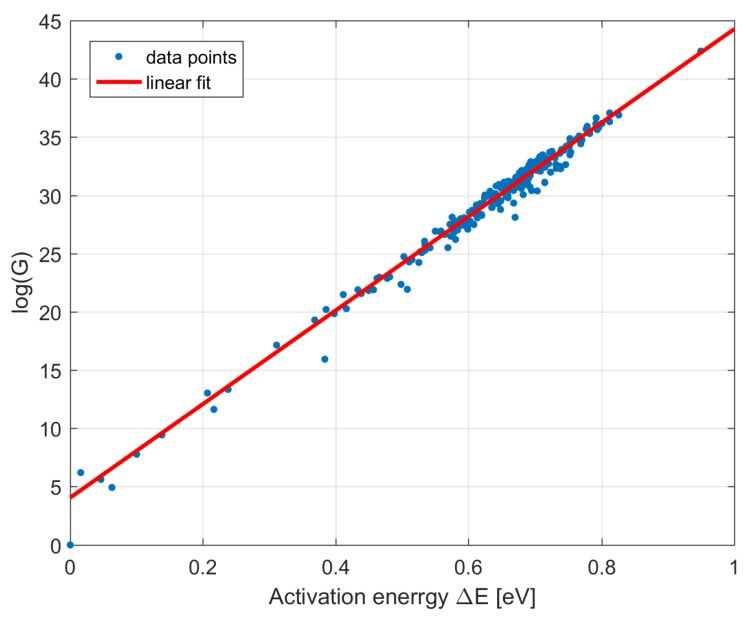
The logarithmic relation between factor *G* and activation energy ΔE. The linear fit to the data leads to the estimation of the MNR constant $E_{MN}=25.3$ meV. The data points include the results for the setups collected by all BRITE satellites.

The dark current data collected from BRITE CCDs give the unique chance for investigation of the correctness of the MNR. Using the dependency between previously obtained log(G) and ΔE, the MNR constant $E_{MN}$ was estimated at 24.8 meV. The linear fitting made on such logarithmic dependency is depicted in Figure 10. To the best of our knowledge, this finding is the first such observation made for PV defects in CCD sensors irradiated in a space environment. For comparison, the value reported for ground-based CCDs (not exposed to irradiation), containing defects mainly in the form of impurities induced during the sensor’s production, was $E_{MN}=25.3$ meV [7].

## 5. Summary

Although the pioneering mission of the BRITE nanosatellites allows one to perform photometric measurements of stars with very high precision, the CCD sensors installed onboard are exposed to strong irradiation and experience gradual degradation. In this paper we present a detailed analysis of the progress of the dark current generation in the BRITE CCDs. Several steps of image processing and proper data normalization were implemented to retrieve useful information about the evolution of thermal activity in pixels.

The results obtained from the analysis of 0.14% of CCD pixels show clearly a linear increase of the number of defects induced in CCDs of all five nanosatellites. When compared with unshielded satellites, the special polyethylene-based shielding installed onboard BRITE Heweliusz (BHr) managed to reduce the amount of inflicted damage by a factor of two. Moreover, the two satellites equipped with full (BHr) or partial (BTr) shielding made of Borotron or tungsten, respectively, successfully protected sensors against the radiation during the launch. Unfortunately, the partial shielding in BTr reduced the amount of defects created in the orbit only by 25%. It is still an unresolved issue why one of the unshielded satellites—BAb— shows nearly 50% larger progress in CCD degradation when compared with the remaining unshilded devices.

Investigations of temperature dependencies of dark current revealed that the most probable type of defects in BRITE sensors is the phosphorus-vacancy (PV) pair. The obtained activation energy of the thermal process at 0.44 eV below the silicon conduction band agrees with the previous reports of researchers examining proton-irradiated CCDs. Eventually, the empirical Meyer-Neldel rule (MNR) for the dark current was confirmed in our data. The MNR constant $E_{MN}$ was estimated at 24.8 meV which is the first such report from CCDs working in space and containing PV defects.

The measured progress of the number of appearing defects may be valuable information for future nanosatellite missions which will meet similar size, weight and power constraints. Moreover, the results of the presented analysis are essential for assessing the usefulness of relatively small shielding made of tungsten or Borotron. The identification of PV defects allows for considering possible ways of treating the CCD sensors via annealing.

## Figures and Tables

**Table 1:** Characteristics of CCDs installed in BRITE nanosatellites.

Parameter	Value
CCD type	interline
Number of pixels	4072×2720
Dimensions	37.25 mm × 25.7 mm
Pixel size	9 μm × 9 μm
Saturation charge	90,000 e${}^{-}$
Bit resolution	14 bit

**Table 2:** Increase of dark current in BRITE CCDs.

Satellite Name	Shielding (Material)	Dark Current Growth		
		[$e^{-}s^{-1}{\mathrm{pix}}^{-1}{\mathrm{year}}^{-1}$]		
		@15 ${}^{\circ }$C	@25 ${}^{\circ }$C	@35 ${}^{\circ }$C
BHr	yes (Borotron, 10 mm)	786	2846	6079
BTr	partial (tungsten 2 mm)	1202	4134	9365
UBr	no	1372	5318	12,095
BLb	no	1363	5167	12,076
BAb	no	1851	7535	17,975
